# Changes in synaptic markers after administration of ketamine or psychedelics: a systematic scoping review

**DOI:** 10.3389/fpsyt.2023.1197890

**Published:** 2023-06-26

**Authors:** Simon Zhornitsky, Henrique N. P. Oliva, Laura A. Jayne, Aza S. A. Allsop, Alfred P. Kaye, Marc N. Potenza, Gustavo A. Angarita

**Affiliations:** ^1^Department of Psychiatry, Yale University School of Medicine, New Haven, CT, United States; ^2^Clinical Neuroscience Research Unit, Connecticut Mental Health Center, New Haven, CT, United States; ^3^Connecticut Mental Health Center, New Haven, CT, United States; ^4^Clinical Neurosciences Division, VA National Center for PTSD, West Haven, CT, United States; ^5^Child Study Center, Yale University School of Medicine, New Haven, CT, United States; ^6^Department of Neuroscience, Yale University, New Haven, CT, United States; ^7^Connecticut Council on Problem Gambling, Hartford, CT, United States; ^8^Wu Tsai Institute, Yale University, New Haven, CT, United States

**Keywords:** synaptic density, SV2A, PSD-95, synapsin-1, synaptophysin-1, synaptotagmin-1, dendrite, addiction

## Abstract

**Background:**

Ketamine and psychedelics have abuse liability. They can also induce “transformative experiences” where individuals experience enhanced states of awareness. This enhanced awareness can lead to changes in preexisting behavioral patterns which could be beneficial in the treatment of substance use disorders (SUDs). Preclinical and clinical studies suggest that ketamine and psychedelics may alter markers associated with synaptic density, and that these changes may underlie effects such as sensitization, conditioned place preference, drug self-administration, and verbal memory performance. In this scoping review, we examined studies that measured synaptic markers in animals and humans after exposure to ketamine and/or psychedelics.

**Methods:**

A systematic search was conducted following PRISMA guidelines, through PubMed, EBSCO, Scopus, and Web of Science, based on a published protocol (Open Science Framework, DOI: 10.17605/OSF.IO/43FQ9). Both *in vivo* and *in vitro* studies were included. Studies on the following synaptic markers were included: dendritic structural changes, PSD-95, synapsin-1, synaptophysin-1, synaptotagmin-1, and SV2A.

**Results:**

Eighty-four studies were included in the final analyses. Seventy-one studies examined synaptic markers following ketamine treatment, nine examined psychedelics, and four examined both. Psychedelics included psilocybin/psilocin, lysergic acid diethylamide, N,N-dimethyltryptamine, 2,5-dimethoxy-4-iodoamphetamine, and ibogaine/noribogaine. Mixed findings regarding synaptic changes in the hippocampus and prefrontal cortex (PFC) have been reported when ketamine was administered in a single dose under basal conditions. Similar mixed findings were seen under basal conditions in studies that used repeated administration of ketamine. However, studies that examined animals during stressful conditions found that a single dose of ketamine counteracted stress-related reductions in synaptic markers in the hippocampus and PFC. Repeated administration of ketamine also counteracted stress effects in the hippocampus. Psychedelics generally increased synaptic markers, but results were more consistently positive for certain agents.

**Conclusion:**

Ketamine and psychedelics can increase synaptic markers under certain conditions. Heterogeneous findings may relate to methodological differences, agents administered (or different formulations of the same agent), sex, and type of markers. Future studies could address seemingly mixed results by using meta-analytical approaches or study designs that more fully consider individual differences.

## Introduction

Approximately 20.4 million people in the United States met diagnostic criteria for substance use disorders (SUDs) in 2021 (1). SUDs have been associated with homelessness, incarceration, violence, poor health, and premature death (2–6). Although initially rewarding, chronic use of alcohol and drugs may lead to progressively restricted, habitual, and maladaptive patterns of compulsive seeking and administration of substances (7). The difficulty of treating SUDs is reflected by the lack of medications with indications from the Food and Drug Administration (FDA) for specific types of SUDs (e.g., cocaine and cannabis), while others (e.g., opioids, alcohol, and tobacco) have high relapse rates despite available FDA-approved pharmacotherapies (8–14). Despite their controlled nature and possibility—or concerns—of abuse, ketamine, and psychedelics have potential therapeutic value in treating SUDs and other psychiatric conditions.

The therapeutic potential of ketamine and psychedelics may involve neuronal synaptic plasticity arising from dendritic connections. Originally characterized in the 19th century, dendritic spines are small neuronal protrusions that represent sites of neuronal contact (15). Dendrites on pyramidal neurons can be classified as basal or apical. Basal dendrites are shorter, denser, and receive input from the base of pyramidal cells near the soma, whereas apical dendrites are longer, less dense, and emerge from the neuronal apex (16). A single apical dendrite emerges from the top/apex of a pyramidal neuron and is orientated towards the pial surface. Multiple basilar dendrites emanate from the bottom sides of a pyramidal neuron and extend laterally. Dendritic spine growth occurs as part of synaptic plasticity, which underlies learning phenomena relevant to substance use. This process includes consolidation of memory for the drug along with drug-associated cues and contexts (17), behavioral sensitization (18, 19), conditioned place preference (20, 21), and drug self-administration (22, 23). In cannabis use disorder, verbal memory performance was also shown to be affected (24).

Robinson and Kolb demonstrated that exposure to several drugs of abuse in animals (i.e., amphetamine, cocaine, morphine, nicotine) can alter the number of spines and branches of dendrites in the nucleus accumbens (NAc) and prefrontal cortex (PFC), sometimes in opposite directions (25–28). In addition, animal studies show that synaptic markers can change with intoxication vs. withdrawal (21, 29). In humans with cocaine use disorder (CUD), our group was the first to show lower synaptic markers in the anterior cingulate cortex (ACC), ventromedial PFC, and medial orbitofrontal cortex (OFC), compared to control subjects without CUD (30). Furthermore, individuals with cannabis use disorder were shown to have lower synaptic markers in the hippocampus (24). Drug-induced structural plasticity may underlie protective adaptations to addiction or promote addictive behaviors such as compulsive drug use, craving, and vulnerability to relapse despite sustained abstinence (28). Synaptic proteins can be quantified in both humans and in animal models, offering an indirect measure of both pre-synaptic (e.g., synapsin-1, synaptophysin-1, synaptotagmin-1, SV2A) (31–35) and post-synaptic [e.g., post-synaptic density protein-95 (PSD-95)] (36) function. Synaptic protein markers have been studied in conjunction with dendritic structural changes (37–41)—the growth of dendritic spines can induce precise, synapse-specific effects which affect behavior (24, 42–44). Critically, a gap exists in our understanding of how synaptic protein densities change with psychedelics and drugs of abuse which may facilitate improved treatment of SUDs (12).

Ketamine and psychedelic agents [e.g., psilocybin, lysergic acid diethylamide (LSD), and ibogaine] induce structural plasticity and offer a promising avenue for improving addiction-related outcomes (45). Ketamine and psychedelics have been hypothesized to help shift maladaptive behavioral patterns in addictions, possibly because they can facilitate “transformative experiences” or “spiritual awakenings”. During these transformative experiences individuals report enhanced states of awareness that can produce lasting positive effects on subjective well-being, social attitude, and perceived meaning in life (46–49). Preclinical studies show that these psychoactive substances can modify spine density (50–53) or synaptic proteins (54–58) in brain structures that are involved in cognitive control as well as learning and memory. Despite abuse liability, data suggest potential beneficial effects of ketamine and psychedelics in the treatment of SUDs caused by alcohol (59–62), cocaine (63–67) or opioids (68–70). Ketamine-induced spine growth in the PFC appears necessary for its antidepressant effects (71), raising the possibility that structural plasticity may represent a common mechanism underlying ketamine’s therapeutic effects across psychiatric disorders.

Due to the literature suggesting that alterations to synaptic markers are associated with SUD outcomes in preclinical models, we conducted a scoping review wherein we explored preclinical and clinical literature on the association between administration of ketamine and/or psychedelics and the subsequent changes to synaptic markers.

## Methods

This review was performed according to the preferred reporting of items for systematic reviews and meta-analysis extension for scoping reviews (PRISMA-ScR) (72), following the framework proposed by the Joanna Briggs Institute (73).

### Protocol and registration

A protocol was created and published in advance, describing the envisioned search strategy, eligibility criteria, study screening and selection process, and data extraction. The protocol was registered on the Open Science Framework (DOI: 10.17605/OSF.IO/43FQ9) and is available online at: https://osf.io/43fq9/.

### Eligibility criteria

Both *in vitro* and *in vivo* (preclinical and clinical) studies were included. *In vivo* was defined as any study in a living vertebrate animal, inclusive of humans, excluding non-vertebrates. *In vitro* was defined as studies of cell lines, organoids, or biological molecules outside their normal biological contexts. Studies on the following synaptic markers were included: structural dendritic changes (e.g., dendritic spine density, arborization), PSD-95, synapsin-1, synaptophysin-1, synaptotagmin-1, and SV2A. Inclusion criteria were as follows: studies analyzing use of ketamine or psychedelics evaluating synaptic markers and published in English. Exclusion criteria included lack of synaptic marker analysis, combination treatment, animal models not related to stress or addiction (e.g., neuronal injury, schizophrenia, dyskinesia), use of immune cells, and use of transgenic animals or genetic strains with significant health abnormalities that could affect synaptic markers (e.g., thyroid dysgenesis) (74). We did not include spine shape classifications in our analyses since they are based on arbitrary criteria and prone to bias (75). Finally, although there are data on structural neural plasticity with non-hallucinogenic psychedelics (e.g., tabernanthalog) (50), the present review did not include those here, given their lack of capacity to induce altered perception, which is in line with the definition of a psychedelic (76).

### Information sources and search strategy

The search was performed through PubMed, EBSCO, Scopus, and Web of Science up to January 01, 2023. Grey literature was not considered. The search strategy encompassed the concepts of “synaptic density,” “ketamine,” and “psychedelics,” using the terms psilocybin, psilocin, lysergic acid diethylamide, LSD, N,N-Dimethyltryptamine, DMT, mescaline, ibogaine, ayahuasca, 2,5-dimethoxy-4-iodoamphetamine, DOI, ketamine, synaptic density, SV2A, synapsin, synaptotagmin, synaptophysin, PSD 95, dendrit*. The search was applied and adapted according to required syntax for each database. For example, the following search applied to PubMed.

(psilocybin[tiab] OR psilocin[tiab] OR “lysergic acid diethylamide”[tiab] OR LSD[tiab] OR N,N-dimethyltryptamine[tiab] OR DMT[tiab] OR mescaline[tiab] OR ibogaine[tiab] OR ayahuasca[tiab] OR 2,5-Dimethoxy-4-iodoamphetamine[tiab] OR DOI[tiab] OR ketamine[tiab]) AND (“synaptic density”[tiab] OR SV2A[tiab] OR synapsin[tiab] OR synaptotagmin[tiab] OR synaptophysin[tiab] OR “PSD 95”[tiab] OR dendrit*[tiab])

The search with their respective results is presented in Supplementary material. Duplicates were removed with aid of EndNote 20 (Clarivate Analytics, Philadelphia, Pennsylvania, United States). The search was also conducted using the term “synap*” instead of “synaptic density.” Even though the number of papers retrieved was higher, the number of studies included did not change.

### Selection of sources of evidence

For study selection, authors SZ and HO participated in the searching and screening of papers. For studies in which the two reviewers did not reach agreement, a third reviewer was consulted (GA). The screening was performed in two stages. Titles and abstracts were screened first, followed by a full-text screening during the second stage. If the papers met inclusion criteria in stage one, they were moved forward to the stage two. If they did not meet inclusion criteria in either stage, they were excluded.

### Data charting process and data items

The data were extracted to a table, with the following information: author, year of publication, agent, dose, route of administration, duration, *in vivo* or *in vitro*, animal, line, sex, region, synaptic marker(s), method, time between last administration and evaluation, main outcomes, model (basal and/or stress) and paradigm (administration pre-, mid-, or post-stress) (Supplementary Table S1).

## Results

**Figure 1:**
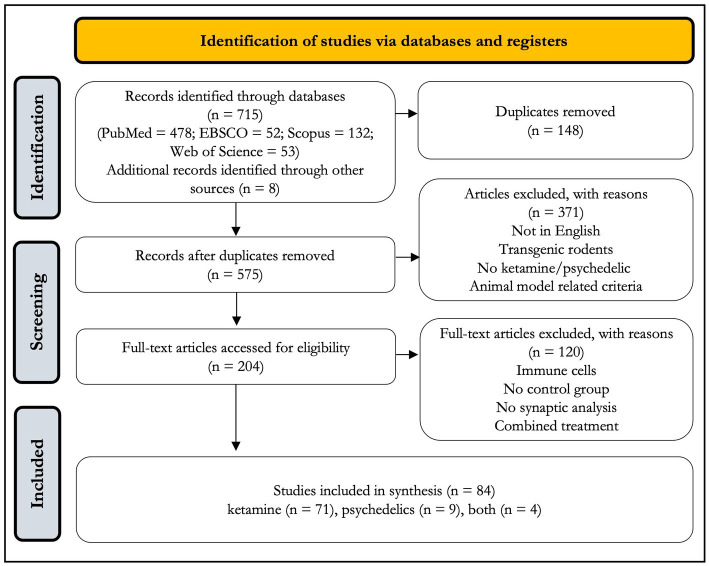
Flow diagram showing inclusion and exclusion strategy.

Eighty-four studies were included in the final analysis (Table 1 and Figure 1). Seventy-one studies examined synaptic markers following ketamine treatment, nine examined psychedelics, and four examined both. All were conducted exclusively in animals, except for four (77–80).

**Table 1:** Summary of included studies.

	Ketamine	Psychedelics
Agents	Racemic ketamine (*n* = 65), R-ketamine (*n* = 5), S-ketamine (*n* = 5)	DMT (*n* = 2), DOI (*n* = 7), ibogaine/noribogaine (*n* = 2), LSD (*n* = 3), psilocyn/psilocybin (*n* = 3)
*In vivo* or *in vitro*	*In vivo* (*n* = 63), *in vitro* (*n* = 14)	*In vivo* (*n* = 8), *in vitro* (*n* = 6)
Subjects	Rats/rat cells (*n* = 33), mice/mouse cells (*n* = 40), humans/human cells (*n* = 4), non-human primates (*n* = 1)	Rats/rat cells (*n* = 9), mice/mouse cells (*n* = 3), pigs (*n* = 1)
Brain regions	Cortical (ACC, dlPFC, FC, IL, mPFC, OFC, PFC, PrL, vmPFC; *n* = 55), hippocampal (CA1, CA3, DG; *n* = 45), striatal (NAc shell and core, dorsal striatum; *n* = 11)	Cortical (FC, mPFC, IL, PFC, PrL, OFC; *n* = 11), hippocampal (CA1, CA3, DG; *n* = 4)
Synaptic markers	PSD-95 (*n* = 36), SV2A (*n* = 1), SYN (*n* = 20), SYP (*n* = 3), SYT (*n* = 1), structural dendritic measures (*n* = 35)	PSD-95 (*n* = 3), SV2A (*n* = 1), SYN (*n* = 1), structural dendritic measures (*n* = 11)
Overall outcome summary^a^	↑ (≈50%), — (≈27%), ↓ (≈23%)	↑ (≈47%), — (≈40%), ↓ (≈13%)

The total counts differ from 84, because some of the studies used both *in vivo* and *in vitro* approaches, and/or investigate more than one agent and/or more than one type of animal. DMT, dimethyltryptamine; DOI, 2,5-Dimethoxy-4-Iodoamphetamine; LSD, lysergic acid diethylamide; ↑, increase; —, no change; ↓, decrease; ACC, anterior cingulate cortex; CA1 and CA3, hippocampal subregion cornu ammonis; DG, hippocampal subregion dentate gyrus; FC, frontal cortex; NAc, nucleus accumbens; dlPFC, dorsolateral prefrontal cortex; IL, infralimbic; mPFC, medial prefrontal cortex; PFC, prefrontal cortex; vmPFC, ventromedial prefrontal cortex; PrL, prelimbic; OFC, orbitofrontal cortex; SYN, synapsin-1; SYP, synaptophysin-1; SYT, synaptotagmin-1; SV2A, synaptic vesicle glycoprotein 2A.

a Approximate percentages within included studies.

A complete list of study characteristics is presented in Supplementary Table S1.

### Ketamine

#### Ketamine administration *in vitro*

Fourteen studies have examined ketamine’s effect on synaptic markers *in vitro*, and there was no consistent pattern of outcomes observed across these studies. Instead, different results were found for synaptic markers based on the dosages used and the evaluation times. In rat hippocampal neurons, ketamine (2 μM) did not change spine density or colocalization with synapsin-1 after 1 h of treatment, whereas it increased colocalization of spines with synapsin-1 (suggesting increased presynaptic contacts) after 24 h of treatment (81). However, another study found synapsin-1 was dose-dependently reduced by S-ketamine (3–25 μM) in rat hippocampal neurons (82). There is also evidence that ketamine (100 μM) lowered phosphorylated synapsin (P-S9-synapsin), without affecting synapsin-1 in mouse PFC neurons, suggesting increased presynaptic release potential (83). At lower doses, ketamine (0.1–10 μM) increased dendritic arbor complexity, spine density, and synaptic markers [as measured by colocalization of PSD-95 and vesicular glutamate transporter 1 (VGLUT1)] in rat cortical neurons, mouse mesencephalic dopaminergic neurons, and human pluripotent stem cell-derived dopaminergic neurons (38, 39, 50, 77). Additionally, 4 days of ketamine (100 μM) reversed reduction of PSD-95 expression and spine density in response to 4 days of dexamethasone exposure in primary rat hippocampal cultures (41). By contrast, ketamine (100–500 μM) decreased spine density and/or synaptophysin puncta per μM of dendrites in both rat cortical and hippocampal neurons as well as human striatal projection neurons (79, 80, 84, 85). Finally, in rat GABAergic neurons, ketamine reduced the number of dendritic branching points at higher doses (10 and 20 μg/mL) when treated for 1 h, or at much lower doses (0.01, 0.1 and 1 μg/mL) when neurons were treated for up to 96 h (86, 87).

#### Single-dose ketamine administration *in vivo*

Thirty-two studies have examined effects of administration of a single dose of ketamine *in vivo*. Thirty-one such studies involved rodents, and one included monkeys and human subjects. In rodent studies, 0.1–5 mg/kg of ketamine had no effect on PSD-95, synapsin-1, dendritic spine density, or branching in the hippocampus or PFC (88–93). On the other hand, ketamine (1 mg/kg) increased hippocampal PSD-95 and dendritic spine density (94, 95). Interestingly, analysis of sex differences showed that ketamine at 5 mg/kg increased PSD-95 and synapsin-1 in PFC among male, but not female rodents, although neither 2.5 or 5 mg/kg changed spine density (40).

Studies have examined effects of ketamine on both apical and basal dendrite synaptic markers. Ketamine (7.5 mg/kg) increased apical spine markers within the hippocampal CA1 region (96). Furthermore, ketamine (10 mg/kg) increased apical spine density, PSD-95, and synapsin-1 in the mPFC (79, 97–101). Regarding basal spines, ketamine (10 mg/kg) increased spine density in the prelimbic cortex (99). The same dose of ketamine also increased apical spine density in the cerebral cortex (85) and overall spine density in the PFC (38).

In other studies, ketamine (10 mg/kg) did not change PSD-95 levels in the OFC and hippocampus, but it reduced PSD-95 phosphorylation on Thr-19 in hippocampal membranes, suggesting decreased GluA1 receptor internalization (102, 103). Ketamine (10 mg/kg) also increased PSD-95 levels and mPFC post-synaptic density while decreasing PSD-95 levels and hippocampal post-synaptic density. Interestingly, analysis of the homogenate showed ketamine decreased PSD-95 in the mPFC but had no effect in the hippocampus (57). In another study, S-ketamine (10 mg/kg) decreased synapsin-1 level in the hippocampus (104). At a higher dose, S-ketamine (15 mg/kg) increased hippocampal synapsin-1 expression, but decreased synaptotagmin-1 expression (105). In addition, 15 mg/kg of ketamine increased dendritic spine density and arborization in dorsolateral striatal spiny projection neurons (106). At 25 and 50 mg/kg of ketamine, PSD-95 mRNA expression was reduced in the dorsomedial striatum (107). At 150 mg/kg of ketamine, PSD-95 expression was increased in the cerebral cortex (108).

Pregnant rodents that received ketamine (intravenous infusion at a rate of 40–60 mg/kg/h for 2–3 h, or 200 mg/kg infusion for 3 h) produced offspring with reduced dendritic spine density, arborization, and branch number as well as reduced PSD-95, synapsin-1, and synaptophysin-1 in the hippocampus (109–112). The PFC showed increased dendritic spine density, arborization, branch number, and PSD-95, but reduced synaptophysin-1 in the offspring of rodents who were administered ketamine (mean infusion dose = 144 mg/kg over two hours) during pregnancy (113).

Ketamine (0.5 mg/kg) administered to monkeys and humans with major depression/post-traumatic stress disorder (PTSD) did not change SV2A binding 24 h later in the dorsolateral prefrontal cortex (dlPFC), ACC, or hippocampus, as measured with 11C-UCB-J positron emission tomography (PET) imaging (78). However, a post-hoc analysis showed that lower SV2A binding at baseline was associated with ketamine-induced increases in SV2A binding in these regions in humans.

#### Repeated ketamine administration *in vivo*

Eight studies in rodents have examined repeated administration of ketamine. At a low dose (0.5 mg/kg; once daily for 11 days) hippocampal synapsin-1 expression was increased (54). In the NAc, ketamine self-administration (0.5 mg/kg/infusion; 3 days a week) for 4 weeks had no effect on spine density (114). When sex differences were considered, ketamine (2.5 and 5 mg/kg; once a week for 7 weeks) was associated with spine density increases among male rodents in the NAc shell, but not in the NAc core, whereas among females, only the 5 mg/kg dose increased spine density in the NAc shell and core (115). Additionally, 10 mg/kg of ketamine (once daily for 21 days) increased hippocampal synapsin-1 expression among males, but not females (116). Finally, ketamine (5–80 mg/kg; daily 5–14 days) decreased dendritic spine density, arborization, PSD-95 expression, and/or post-synaptic density thickness in the hippocampus, dorsal striatum, and/or vmPFC (117–120).

#### Ketamine effects in animal models of stress *in vivo*

Eight studies have examined ketamine under chronic unpredictable stress (CUS). One study found that CUS did not change PSD-95 or synapsin-1 levels in the PFC, and these markers remained unchanged following ketamine (1 mg/kg) post-treatment (93). However, ketamine pre-treatment (3 mg/kg) or post-treatment (10 mg/kg) increased spine density and/or arborization in the PFC and hippocampal CA1 among resilient rodents and increased spine density in the CA3 among rodents showing anhedonic behavior in the sucrose preference test following CUS exposure (75, 121). Additionally, post-treatment administration of racemic or S-ketamine (10–20 mg/kg) reversed CUS-induced deficits in dendritic spine density, PSD-95, post-synaptic density thickness, and synapsin-1 in the hippocampus and/or PFC (56, 122–125).

Six studies have examined ketamine and chronic corticosterone. Two studies did not find ketamine post-treatment (0.1–1 mg/kg) to effect PSD-95 or synapsin-1 in the hippocampus and PFC of rodents treated chronically with corticosterone (89, 126). By contrast, four studies found that ketamine (1–5 mg/kg) pre- and/or post-treatment ameliorated corticosterone-reduced dendritic arborization, PSD-95, and/or synapsin-1 in the hippocampus (92, 94, 95, 127).

Five studies have examined ketamine with social defeat stress (SDS). SDS decreased dendritic spine density and PSD-95 in the hippocampal DG and CA3 regions and prelimbic cortex/PFC. These effects were reversed by 10 mg/kg ketamine post-treatment with racemic as well as R- and S-ketamine formulations (128–132). By contrast, no form of ketamine altered increased spine density and PSD-95 in the NAc caused by SDS (128, 129, 132).

Four studies have examined ketamine under chronic restraint stress (CRS). Pre-treatment with racemic or R-ketamine (5–10 mg/kg) reversed CRS-induced PSD-95 deficits in the PFC (91, 133). Ketamine post-treatment reversed CRS-induced decreases in synaptophysin-1 in the hippocampus and PFC, with sub-anesthetic doses not reported (134). However, another study found CRS did not lead to hippocampal dendritic spine density decreases and ketamine (10 mg/kg) had no effect when given post-treatment daily for 3 days (135).

Four studies have examined ketamine with the novelty-suppressed feeding test (NSF). Ketamine (0.1–1 mg/kg) administered at the end of 24 h of food deprivation did not change hippocampal PSD-95 or synapsin-1 (136, 51) or spine density (137) in rodents that subsequently underwent NSF participation. Contradictory to these results other studies showed ketamine (1 mg/kg) administration increased hippocampal PSD-95 and synapsin-1 expression (138), as well as dendritic spine density in the same paradigm (51).

Three studies have examined ketamine with foot-shock stress. Ketamine (10 mg/kg) post-treatment reversed decreases in PSD-95 and synapsin-1 in the PFC induced by inescapable foot-shock stress (97). In another study, ketamine (10 mg/kg) post-treatment reversed decreased PSD-95 in the PFC induced by exposure to foot-shock stress conditioning but did not affect increased PSD-95 in the amygdala (139). Finally, acute foot-shock stress did not alter dendritic spine density in the PFC, and this measure did not change after ketamine (10 mg/kg) post-treatment (140).

Two studies have examined ketamine in tail-suspension and open-field tests. Ketamine (0.1 mg/kg) pre-treatment had no effect on dendritic spine density, PSD-95, or synapsin-1 in the hippocampus or PFC (88, 90).

One study has examined ketamine with chronic intermittent cold stress (CIC). Ketamine (10 mg/kg) post-treatment reversed CIC-induced increases in PSD-95 levels in the OFC (103).

One study has examined ketamine with social isolation (SI). Ketamine (5 mg/kg) post-treatment reversed SI-induced reductions in PSD-95, synapsin-1, and dendritic spine density in the PFC of male rodents. Ketamine did not affect synaptic markers following SI in the PFC of female rodents (40).

One study has examined ketamine with respect to lipopolysaccharide administration. R-ketamine (10 mg/kg) post-treatment reversed lipopolysaccharide reductions in dendritic spine density in the prelimbic cortex and hippocampal CA3 and DG regions (141).

### Psychedelics

Seven studies have examined 2,5-dimethoxy-4-iodoamphetamine (DOI); four were conducted *in vitro* and three *in vivo*. The *in vitro* studies that administered 1–3 μM of DOI found no effect on spine density in cortical neurons or on spine density, PSD-95, or synapsin-1 in hippocampal neurons (142–144). At a higher dose of DOI (10 μM) *in vitro*, there were increases in dendritic arbor complexity, dendritic branches, spine density, and synaptic markers (measured via colocalization of PSD-95 and VGLUT1) in cortical neurons (38). *In vivo*, a single-dose of DOI (2 mg/kg) increased spine density in the frontal cortex among rodents with intact 5-HT2A receptors, but not among 5-HT2A-receptor-knockout rodents (145). However, the same dose of DOI had no effect on liposaccharide-induced reductions in spine density in the hippocampus or mPFC (141). Finally, *in vivo* treatment with DOI 5 μg/0.5 μL injected directly into the left OFC once a week for 3 weeks reduced dendritic spine density and PSD-95 (146).

Three studies have examined psilocin/psilocybin. One study was conducted *in vitro* and two *in vivo*. Psilocin (10 μM) *in vitro* increased dendritic branches and arbor complexity in cortical neurons (38). *In vivo* studies showed that administration of psilocybin, at doses of 0.08 to 8 mg/kg, increased both PSD-95 and SV2A expression in the PFC and increased SV2A in the hippocampus (55, 58).

Three studies have examined LSD. Two studies were conducted *in vitro* and one *in vivo*. LSD (10 μM) *in vitro* increased dendritic arbor complexity, dendritic branches, spine density, and synaptic markers (measured via colocalization of PSD-95 and VGLUT1) in cortical neurons (38, 39). Moreover, *in vivo* administration of LSD (30 μg/kg) once daily for 7 days increased spine density in the PFC and reversed CRS-induced reductions in spine density in the PFC of rodents when CRS was administered mid-stress (147).

Two *in vitro* studies have examined ibogaine/noribogaine. One study reported noribogaine (10 μM) *in vitro* increased dendritic arbor complexity in cortical neurons while ibogaine did not (38). In the other, both ibogaine and noribogaine (dose not reported) increased dendritic arbor complexity, and ibogaine increased spine density in cortical neurons (50).

Two studies have examined DMT. DMT (90 μM) *in vitro* increased dendritic branches and arbor complexity in cortical neurons. *In vivo* DMT (10 mg/kg) also increased dendritic spines in the PFC (38). On the other hand, *in vivo* DMT (1 mg/kg) every 3rd day for 7 weeks decreased spine density in the PFC of female but not male rodents (148).

**Figure 2:**
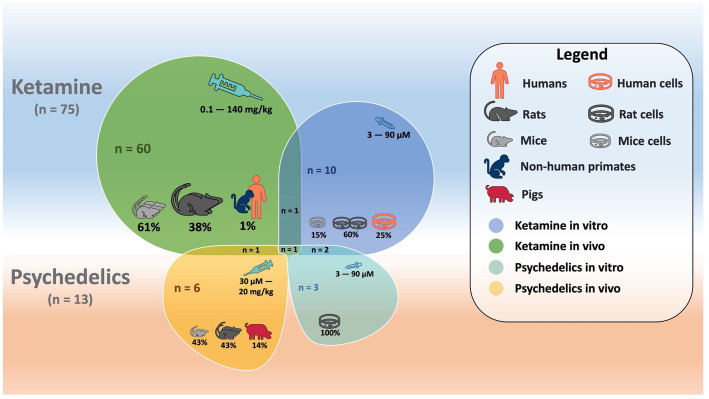
Schematic overlapping shapes illustrating features (i.e., number of studies, cells studied, species, doses, etc.) for *in vitro* and *in vivo* studies investigating ketamine and psychedelics.

A schematic image (Figure 2) illustrates features of included studies investigating ketamine and psychedelics.

## Discussion

We reviewed *in vitro* and *in vivo* studies across species that investigated effects of ketamine or psychedelics on synaptic markers. Data suggest heterogenous findings when ketamine was administered under basal conditions. However, ketamine consistently prevented or reversed stress-induced reductions in synaptic markers in the hippocampus and/or PFC. Existing studies suggest that some psychedelics (e.g., LSD and psilocybin) induce structural plasticity in prefrontal cortical dendrites, although further studies are needed.

Structural plasticity effects can vary by brain region, marker, and behavioral task for ketamine and psychedelics, in some ways reflecting what has been reported for substance use. Studies of substance use suggest mixed results relating to synaptic markers depending on subregions of the brain investigated, whether drugs are experimentally delivered vs. self-administered, and the species being studied (24, 28, 30, 149, 150). Protein quantification (e.g., PSD-95) (57), dose timing (79, 86, 87), and dose-dependent effects (117, 118, 120) may all influence synaptic markers. For example, repeated and/or high-dose treatment with ketamine leads to persistent depression of glutamatergic signaling and prevention of synaptogenesis (84, 107, 117, 118), unlike single treatment with lower doses which can enhance plasticity, particularly when measured with two-photon longitudinal imaging [e.g., (97–99, 101)]. Ketamine’s ability to reduce synaptic markers at high doses may not be surprising since a meta-analysis showed that single-dose ketamine produces dose- and plasma-level-dependent cognitive impairment (151). Regarding psychedelics, dose effects were examined in one study with psilocybin, but no clear dose-response was observed (55). Too few studies exist per agent to make definitive conclusions regarding dose effects of psychedelics.

The type of agent administered (or different formulations of the same agent) is another potential confounding factor between studies. In general, LSD was the most consistently effective at increasing synaptic markers (38, 147, 39), whereas the data for DOI were the least robust. The ability of psychedelics to increase levels of synaptic markers is believed to be related to activation of 5-HT2A receptors. Stimulation of these receptors leads to enhancement of membrane excitability, most notably in proximal apical dendrites (152). Further support for this hypothesis stems from data showing that the selective serotonergic 5-HT2A/2C antagonist ketanserin completely blocked the ability of LSD, DMT, and DOI to promote both neuritogenesis and spinogenesis (38). Also, DOI enhancement of spine density was absent in 5-HT2A-receptor-knockout animals (145). Interestingly, of psychedelics, LSD has the highest affinity for 5-HT2A receptors, which may explain its effectiveness at increasing synaptic markers (153, 154).

Differences across studies may also be explained by different formulations of ketamine. Blocking N-methyl-D-aspartate (NMDA) receptors with ketamine leads to increased release of glutamate, increased α-amino-3-hydroxy-5-methyl-4-isoxazolepropionic acid (AMPA) activity / receptor expression (155–159), activation of mammalian target of rapamycin complex 1 (mTOR1) (41, 54, 102), and brain-derived neurotrophic factor (BDNF) (41, 115, 129, 132, 138). S-ketamine leads to a maximum plasma level concentration approximately three-fold greater and a binding affinity for NMDA receptors that is approximately five-fold greater than R-ketamine (160, 161).

Sex may influence the effects of ketamine and DMT on synaptic markers. Intriguingly, many studies wherein ketamine failed to alter synaptic markers were conducted solely in females (78, 93, 126, 137, 142). Among studies that included and compared both sexes, a decrease in PFC spine density due to administration of micro-doses of DMT was observed only among female rodents (148). Additionally, a ketamine-induced increase in synaptic markers was found only among male rodents in the hippocampus (116) and PFC (40). Moreover, ketamine administration elevated synaptic markers among females in the NAc shell and core, whereas males exhibited elevations only in the shell (115). Previous studies have shown that levels of male and female sex hormones are positively associated with synaptic markers, which may in part explain sex differences observed across studies (162, 163). Taken together, these studies indicate that the same drug exposure can have different effects on synaptic markers in males vs. females. Thus, sex should be taken into consideration in future studies.

The type of marker is another important confounding variable in determining potential effects of ketamine and/or psychedelics on synaptic markers. Not all markers consistently show changes in the same direction. In some studies, structural dendritic changes are not accompanied by changes in protein markers, and *vice*-*versa* (51, 96), while other studies show opposite changes in specific protein markers (104, 113). It is possible that phosphorylation or colocalization of protein markers may be altered without changes to protein expression, but not many studies have examined these variables (38, 39, 83, 102). In addition, some presynaptic markers have been shown to be altered by ketamine, but not by psychedelics, such as synapsin-1 and synaptophysin-1 (55, 144). PSD-95 is a post-synaptic marker of synaptic density that can be changed by ketamine, but there is less evidence for psychedelics (55, 146). PSD-95 regulates synaptic expression and transmission of glutamatergic NMDA and AMPA receptors, which may be the reason behind the ability of ketamine to alter expression of this protein since ketamine works directly via activity at the NMDA receptor (164, 165). SV2A is a synaptic vesicle protein that regulates release of neurotransmitters via action potentials (166) and the only marker that is available to be imaged *in vivo* with PET imaging using the radioligand 11C-UCB-J in humans (167). Studies have validated SV2A as an alternate marker (to synaptophysin-1) of synaptic density (166). Recent clinical translational studies have documented for the first time decreases in the synaptic marker, SV2A, among persons with cocaine and cannabis use disorder (24, 30). In the studies reviewed herein, psilocybin increased SV2A under basal conditions in pigs (58), whereas ketamine increased SV2A only among humans with depression/PTSD who had low baseline SV2A expression in the hippocampus and PFC (78). Another clinical paper showed that among persons with stress-related mood and anxiety disorders, SV2A expression in the dlPFC was negatively associated with measures of worry and tension/anxiety (168). These data are aligned with other findings showing that excess glucocorticoids/stress negatively impacts spine density, which can be reversed by ketamine (41, 56, 92, 94, 95, 130, 169) and LSD (147). Interestingly, across several studies in which stress exposure did not lead to changes in synaptic markers, ketamine did not influence synaptic markers either (51, 88–90, 93, 126, 135, 140, 136). Future studies should examine changes to SV2A alongside other markers to better elucidate relationships between them when exposed to ketamine and psychedelics.

## Limitations

The present review has several strengths and limitations. It is the first to systematically examine changes to synaptic markers following administration of ketamine and psychedelics. Another strength is the inclusion of both *in vivo* and *in vitro* studies. Examining animals *in vivo* allows access to outcomes based on multiple components of the living organism, whereas measuring effects by *in vitro* assays may aid to better control for potentially confounding variables (170). We did not see a consistency within species between *in vivo* and *in vitro* studies when the treatment was comparable, but we cannot exclude this possibility since most studies utilized rodents. The inclusion of multiple synaptic markers (protein and structural) is another strength because it provides more comprehensive characterization of their associations with administration of ketamine or psychedelics. Although the inclusiveness of multiple markers and methodologies is a strength, at the same time, particular factors of each marker (i.e., level of detectability, etc.) may explain heterogenous findings. In particular, studies that have used two-photon longitudinal imaging to examine structural dendritic changes have repeatedly shown increases in synaptic density in response to single-dose administration of ketamine *in vivo* (97–99, 101); however, studies that have used other methods to measure dendritic branching or measured protein synaptic markers have shown mixed results, perhaps relating to the complex relationship between proteins, synaptic density, and dendritic architecture. Even though the pattern in which single administration of ketamine/psychedelics results in enhancement of synaptic markers (when measured with two-photon longitudinal imaging) is not well represented in the current results (Supplementary Table S1), this may in part reflect our inclusion/exclusion criteria. For instance, multiple studies using two-photon longitudinal imaging have shown increased synaptic markers using ketamine/psychedelics, but these were not included because they used transgenic animals, which were excluded in the present review (52, 71, 171, 172). A further limitation is that some of the markers reviewed herein are indirect estimates of synaptic density (166, 173, 174) and may only reflect synaptic density alterations to the extent that the gap between different amounts of these proteins within synapses and the actual (i.e., direct) number of synapses is small. Another limitation is that studies reviewed are not in the context of exposure to non-ketamine/non-psychedelic drugs of abuse (i.e., cocaine, opioids, etc.). Thus, the current findings cannot be generalized to such circumstances. There were also few studies directly comparing psychedelic drugs, which limits conclusions about their effects on synaptic markers. Pertaining to regions of interest, most studies have examined ketamine or psychedelic effects on synaptic density or proteins in the hippocampus and/or PFC. Other brain areas such as the striatum, NAc, and OFC may be implicated, but further research is needed to investigate. Finally, associations between microarchitecture, function, cognition, and behavior are not exclusive to quantification of dendrites or spines but also the morphology of each spine. Strong synaptic connections are formed by spines with large heads, which are stable and express large numbers of glutamatergic AMPA receptors, whereas weak, unstable synaptic connections are formed by spines with small heads (175). Here, we did not examine this outcome, which may limit our interpretation of dendritic structural changes.

## Conclusion

In the present systematic scoping review, we examined potential effects of ketamine and psychedelics on synaptic markers under basal conditions and stress. The results indicate that, when administered once or repeatedly under basal conditions, ketamine produces mixed results in the hippocampus and PFC, regions implicated in the effects of drugs of abuse. The results for psychedelics also show that they can enhance synaptic markers under basal conditions and reverse deficits associated with stress, but the numbers of studies per agent is low. Some of the null or negative findings relating to ketamine and/or psychedelic effects on synaptic markers may be due to methodological differences, agents administered (or different formulation of the same agent), sex, and/or types of markers. Results also suggest that ketamine may produce more robust results when administered before or after stress to prevent or reverse deficits in synaptic markers in the hippocampus and PFC. Decreased synaptic markers in the hippocampus and PFC may be related to reduced tendencies/abilities to regulate emotion and behavior (176–178), while increases in the striatum may signal increased drug-seeking behavior and behavioral sensitization (19, 22, 179). “Normalization” of dysregulated levels of synaptic markers in some of these brain regions may underlie potential benefits of ketamine and psychedelics in the treatment of SUDs. Further research is required to elucidate relationships between changes to synaptic markers after administration of ketamine or psychedelics and improvements in SUD outcomes.

## Author contributions

SZ, HO, LJ, AK, and GA made substantial contributions to the conception or design of the work, or the acquisition, analysis, or interpretation of data for the work. SZ, HO, LJ, AA, AK, MP, and GA made substantial contributions drafting the work or revising it critically for important intellectual content. SZ, HO, and GA agree to be accountable for all aspects of the work in ensuring that questions related to the accuracy or integrity of any part of the work are appropriately investigated and resolved. All authors contributed to the article and approved the submitted version.

## Funding

Funding was provided by R21DA046030-02 (GA), R01DA052454-02 (MP, SZ, HO, and GA), 5P30DA046345-04 (GA and HO), BBRF NARSAD Young Investigator Award (AK), and NIMH K08 MH122733-01 (AK), which covered percent effort for several co-authors.

## Conflict of interest

AK receives or has received research funding from Transcend Therapeutics and Freedom Biosciences, and has filed a provisional patent for combination psychedelic pharmacotherapies in PTSD.

The remaining authors declare that the research was conducted in the absence of any commercial or financial relationships that could be construed as a potential conflict of interest.

## Publisher’s note

All claims expressed in this article are solely those of the authors and do not necessarily represent those of their affiliated organizations, or those of the publisher, the editors and the reviewers. Any product that may be evaluated in this article, or claim that may be made by its manufacturer, is not guaranteed or endorsed by the publisher.
