# Supplementary material for: Changes in synaptic markers after administration of ketamine or psychedelics: a systematic scoping review
Source: Front Psychiatry. 2023 Jun 26;14:1197890. doi: 10.3389/fpsyt.2023.1197890 (PMC10331617; doi:10.3389/fpsyt.2023.1197890)
Supplement: Supplementary file 1 [file Table_1.docx]

Supplementary Material

**Changes in synaptic markers after administration of ketamine or psychedelics: a systematic scoping review**

**Simon Zhornitsky, PhD; Henrique N. P. Oliva, MD, PhD; Laura A. Jayne, MD; Aza S. A. Allsop, MD, PhD; Alfred P. Kaye, MD, PhD; Marc N. Potenza, MD, PhD; Gustavo A. Angarita, MD^*^**

*** Correspondence:** Gustavo A. Angarita, MD: gustavo.angarita@yale.edu

Contents

[1 Supplementary Table S1. Detailed characteristics of the included studies. 2](#_Toc135918076)

[2 Supplementary Table S2. Search Strategies for Scoping Review 9](#_Toc135918077)

[3 Supplementary Table S3. PRISMA-ScR Checklist 10](#_Toc135918078)

# Supplementary Table S1. Detailed characteristics of the included studies.

| **Author, year** | **Agent** | **Dose** | **Route** | **Duration** | ***vivo*or*vitro*** | **Animal** | **Line** | **Sex** | **Region** | **Marker** | **Method** | **Time between administration and evaluation** | **Result** | **Model** | **Paradigm** |
| --- | --- | --- | --- | --- | --- | --- | --- | --- | --- | --- | --- | --- | --- | --- | --- |
| **Akinfiresoye & Tizabi 2013** | Ketamine | 0.25, 0.5 mg/kg | IP | 11 days (once daily) | *in vivo* | Rats | Wistar-Kyoto | M | HIP | SYN | western blot | NR | ↑ | Basal | - |
| **Beurel 2016** | Ketamine | 10 mg/kg | IP | Acute | *in vivo* | Mice | WT | M, F | HIP | PSD-95 | western blot | NR | — | Basal | - |
| **Camargo 2019** | Ketamine | 0.1, 1 mg/kg | IP | Acute | *in vivo* | Mice | Swiss | M | HIP | PSD-95, SYN | western blot | 24 hours | —* | NSF | Post |
| **Camargo 2020a** | Ketamine | 1, 5 mg/kg | IP | Acute | *in vivo* | Mice | Swiss | M | HIP, PFC | PSD-95, SYN | western blot | 30 days | —* | CORT | Pre |
| **Camargo 2020b** | Ketamine | 0.1 mg/kg | IP | Acute | *in vivo* | Mice | Swiss | M | HIP, PFC | PSD-95, SYN | western blot | 1 hour | — | TST;  Basal | Pre |
| **Camargo 2021** | Ketamine | 0.1 mg/kg | IP | Acute | *in vivo* | Mice | Swiss | M | HIP, PFC | PSD-95, SYN | western blot | 22 days | — | CORT; Basal | Post |
| **Camargo 2022a** | Ketamine | 0.1 mg/kg | IP | Acute | *in vivo* | Mice | Swiss | M | HIP, PFC | PSD-95, SYN; structural | western blot; Golgi stain | 1, 24 hours;  7 days | — | TST;  Basal | Pre |
| **Camargo 2022b** | Ketamine | 1, 5 mg/kg | IP | Acute | *in vivo* | Mice | Swiss | M | PFC | PSD-95 | western blot | 18 days | ↑* | Restraint stress;  Basal | Pre |
| **Cameron 2019** | DMT | 1 mg/kg | IP | 60 days | *in vivo* | Rats | Sprague-Dawley | M, F | PFC | structural | Golgi stain | NR | —M, ↓F | Basal | - |
| **Cameron 2021** | Ketamine  Ibogaine | 10 μM  NR | NA | 24 h | *in vitro* | Rats | Sprague-Dawley | M, F | Cortex | structural | TPM | NR | ↑ | Basal | - |
| **Cavalleri 2018** | Ketamine | 0.001, 0.1, 1, 10 μM | NA | 1 h | *in vitro* | Mice;  Human | C57BL/6J;  NA | NR | mesencephalic DA neurons;  iPSC-derived DA neurons | structural | Immunofluorescence label | 72 hours | ↑mice (1, 10 μM);  ↑human (1 μM) | Basal | - |
| **Chen 2021** | Ketamine | 20 mg/kg | IP | 14 days (twice daily) | *in vivo* | Mice | ICR | M, F | HIP (CA1) | PSD-95 | Immunofluorescence label | NR | ↓ | Basal | - |
| **De Bartolomeis 2013** | Ketamine | 25 or 50 mg/kg | IP | Acute | *in vivo* | Rats | Sprague-Dawley | M | Cortex, Striatum (dmCP) | PSD-95 | *in situ* hybridization | 1.5 hours | —cortex, ↓dmCP | Basal | - |
| **De Gregorio 2022** | LSD | 30 μg/kg | IP | 7 days | *in vivo* | Mice | C57BL/6N | M | mPFC | structural | Golgi stain | 24 hours | ↑*,↑ | Restraint stress; Basal | Mid |
| **Dong 2017** | Ketamine | 10 mg/kg | IP | Acute | *in vivo* | Mice | C57BL/6 | M | HIP (DG, CA1, CA3), NAc, PFC | PSD-95; structural | western blot; Golgi stain | 8 days | ↑*PFC, DG, CA3; —NAc, CA1 | Social defeat stress | Post |
| **Fraga 2020** | Ketamine | 1 mg/kg | IP | Acute | *in vivo* | Mice | Swiss | F | HIP (DG) | SYN; structural | western blot; Golgi stain | 1 hour | —SYN; ↑spine density (DG); —arborization | NSF;  Basal | Post |
| **Fraga 2021a** | Ketamine | 1 mg/kg | IP | Acute | *in vivo* | Mice | Swiss | F | HIP (DG) | PSD-95, SYN; structural | western blot; Golgi stain | 24 hours | ↑*; — | CORT; Basal | Post |
| **Fraga 2021b** | Ketamine | 1 mg/kg | IP | Acute | *in vivo* | Mice | Swiss | F | HIP (DG) | structural | Golgi stain | 24 hours | ↑* | CORT;  Basal | Post |
| **Hladik 2019** | Ketamine | 7.5 mg/kg | IP | Acute | *in vivo* | Mice | NMRI | M | HIP (CA1) | PSD-95; structural | western blot; Golgi stain | 6 months | —PSD-95, spine density; ↑branching, number | Basal | - |
| **Holmes 2022** | Ketamine | 0.5 mg/kg | IV | Acute | *in vivo* | Monkey  Humans | Rhesus   NA | F  M, F | ACC, dlPFC, HIP | SV2A | PET scan | 24 hours, 1 week | —Rhesus; ↑@24 h for humans with low baseline SV2A in ACC, dlPFC, —HIP | Basal | - |
| **Hou 2022** | S-Ketamine | 15 mg/kg | IP | Acute | *in vivo* | Mice | C57BL/6N | M | mPFC | PSD-95 | western blot; TEM | 4 days | ↑* | Unpredictable stress | Post |
| **Jefsen 2021** | Psilocybin | 0.5, 1, 2, 4, 8, 14, 20 mg/kg | IP | Acute | *in vivo* | Rats | Sprague-Dawley | M | HIP, PFC | PSD-95, SYN, SYP | western blot | 1.5 hours | —HIP; ↑PSD-95 in PFC  (8 mg/kg) | Basal | - |
| **Jiang 2018** | Ketamine | 100, 300, 500 µM | NA | 6 h | *in vitro* | Rats | Sprague-Dawley | NR | HIP | structural | LSCM | NR | ↓(300, 500 µM) | Basal | - |
| **Jones 2009** | DOI | 1 µM | NA | 0.25, 0.5, 0.75, 1 h | *in vitro* | Rats | Sprague-Dawley | F | Cortex | structural | LSCM | NR | — | Basal | - |
| **Krzystyniak 2019** | Ketamine | 3 mg/kg | IP | Acute | *in vivo* | Mice | C57BL/6J | M | HIP, PFC | structural | LSCM | 23 days | ↑* | Unpredictable stress | Pre |
| **Lazarevic 2021** | Ketamine | 100 µM | IP | 0.5 h | *in vitro* | Mice | C57BL/6J | M | Cortex | P-S9-synapsin, SYN | western blot | NR | ↓P-S9-synapsin, —SYN | Basal | - |
| **Lee 2019** | Ketamine | 10 mg/kg | IP | Acute | *in vivo* | Rats | Sprague-Dawley | NR | Amygdala, PFC | PSD-95 | western blot | 24 hours | ↑*amygdala, —PFC | Foot shock stress | Post |
| **Li 2010** | Ketamine | 10 mg/kg | IP | Acute | *in vivo* | Rats | Sprague-Dawley | M | PFC | PSD-95, SYN | western blot | 24 hours | ↑* | Footshock stress;  Basal | Post |
| **Li 2011** | Ketamine | 10 mg/kg | IP | Acute | *in vivo* | Rats | Sprague-Dawley | M | PFC | PSD-95, SYN | western blot | 48 hours | ↑* | Unpredictable stress | Post |
| **Li 2017a** | Ketamine | 200 mg/kg | IV | Acute | *in vivo* | Rats | NR | M, F | HIP (CA1, CA3) | PSD-95, SYN; structural | western blot; Golgi stain | 38 days (8 in utero; 30 post-natal) | ↓ | Pregnant rat | 14 days of gestation (G14) |
| **Li 2017b** | Ketamine | 200 mg/kg | IV | Acute | *in vivo* | Rats | Wistar | M, F | HIP | structural | Golgi stain | 37 days (7 in utero; 30 post-natal) | ↓ | Pregnant rat | 14 days of gestation (G14) |
| **Li 2021** | Ketamine | 1, 10, 50, 100, 500 μM | NA | 1, 8, 24 hours | *in vitro* | Human (iPSCs) | NA | M | Striatum | structural | Immunofluorescence label | 48 hours | ↓(50,100,500 µM) @24 h | Basal | - |
| **Li 2022** | Ketamine | 10, 100 µM | NA | 1, 8, 24 hours | *in vitro* | Human (iPSCs) | NA | F | Striatum | SYP; structural | Immunofluorescence label | 48 hours | ↓spine density; ↓SYP (100 µM) | Basal | - |
| **Liu 2012** | Ketamine | 10 mg/kg | IP | Acute | *in vivo* | Mice | WT | NR | PFC | structural | TPM | 24 hours | ↑ | Basal | - |
| **Liu 2015** | Ketamine | 10 mg/kg | IP | Acute | *in vivo* | Rats | Sprague-Dawley | M | mPFC | structural | TPM | 24 hours | ↑ | Basal | - |
| **Liu 2017** | Ketamine | 10 mg/kg | IP | Acute | *in vivo* | Mice, Rat | C57BL/6, Sprague-Dawley | M | mPFC | PSD-95, SYN | western blot | 1 hour | —PSD-95; ↑SYN | Basal | - |
| **Ly 2018** | Ketamine Psilocin  DOI  LSD  Ibogaine Noribogaine  DMT | 10 µM (all but DMT), 90 µM (DMT); 10mg/kg (ketamine, DMT) | NA  IP | 24 hours  Acute | *in vitro; in vivo* | Rats | Sprague-Dawley | NR | Cortex | structural | LSCM;  Golgi stain | 24 hours | All ↑ dendritic complexity (apart from ibogaine), —ibogaine; ↑ spine density (ketamine, DMT) | Basal | - |
| **Ly 2020** | Ketamine LSD | 10 μM | NA | NR | *in vitro* | Rats | Sprague-Dawley | NR | Cortex | structural | LSCM | 0.25, 1, 6, 24, 72 hours | ↑synapse density @6, 24 h (ketamine);  ↑ synapse density @1, 6, 24 h and spine density @6, 24 h (LSD) | Basal | - |
| **Lyu 2022** | Ketamine | 10 mg/kg | IP | Acute | *in vivo* | Rats | Wistar-Kyoto | M | HIP, PFC | SYP | western blot | 36 hours | ↑* | Restraint stress | Post |
| **Ma 2022** | R-Ketamine | 10 mg/kg | IP | Acute | *in vivo* | Mice | C57BL/6 | M | PFC | PSD-95 | western blot | 9 days | ↑* | Restraint stress | Pre |
| **Mi 2017** | DOI | 3 µM | NA | 15 minutes | *in vitro* | Rats | Sprague-Dawley | NR | Cortex | structural | SDCM | 30 min | — | Basal | - |
| **Muller 2013** | S-Ketamine | 15 mg/kg | IP | Acute | *in vivo* | Rats | Sprague-Dawley | M | HIP | SYN, SYT | western blot | 1, 2, 4 h; 7 days | ↑SYN@2, 4 h, 7 days; ↓SYT@2, 4 h. | Basal | - |
| **Neis 2016** | Ketamine | 1 mg/kg | PO | Acute | *in vivo* | Mice | Swiss | F | PFC | PSD-95, SYN | western blot | 24 hours | — | Unpredictable stress | Post |
| **Neis 2018** | Ketamine | 1 mg/kg | IP | Acute | *in vivo* | Mice | Swiss | F | PFC, HIP | PSD-95 | western blot | 24 hours | — | CORT | Post |
| **Ogundele & Lee 2018** | Ketamine | 30 mg/kg | IP | 5 days | *in vivo* | Mice | C57BL/6 | NR | HIP (CA1, DG) | structural | Golgi stain | 5 days | ↓ | Basal | - |
| **Paredes 2018** | Ketamine | 10 mg/kg | IP | Acute | *in vivo* | Rats | Sprague-Dawley | F | OFC | PSD-95 | western blot | 2 hours | ↓* | CIC; Basal | Post |
| **Pazini 2016** | Ketamine | 1 mg/kg | IP | Acute | *in vivo* | Mice | Swiss | F | HIP | PSD-95 | western blot | 0.5 hours | ↑* | CORT; Basal | Post |
| **Pazini 2020** | Ketamine | 1 mg/kg | IP | Acute | *in vivo* | Mice | Swiss | F | HIP | PSD-95, SYN | western blot | 1 hour | ↑* | NSF | Post |
| **Piva 2021** | Ketamine | 10 mg/kg | IV | Acute | *in vivo* | Rats | Sprague-Dawley | M | HIP, mPFC | PSD-95 | western blot | 24 hours | ↓HIP, ↑mPFC | Basal | - |
| **Pochwat 2022** | Ketamine | 10 mg/kg | IP | 3 days (once daily) | *in vivo* | Mice | C57BL/6J | M | HIP | structural | dil labeling | 24 hours | — | Restraint stress | Post |
| **Qu 2022** | R-Ketamine  DOI | 10 mg/kg;    2 mg/kg | IP | Acute | *in vivo* | Mice | C57BL/6 | M | HIP (CA1, CA3, DG); PFC (IL, PrL) | structural | Golgi stain | 3 hours | ↑HIP, PrL (R-Ketamine); —(DOI) | LPS;  Basal | Post |
| **Raval 2021** | Psilocybin | 0.08 mg/kg | IV | Acute | *in vivo* | Pig | Danish slaughter | F | HIP, PFC | SV2A | PET scan | 1, 7 days | ↑HIP@1 and 7 days; ↑PFC@7 days | Basal | - |
| **de la Fuente Revenga 2021** | DOI | 2 mg/kg | IP | Acute | *in vivo* | Mice | C57BL/6 | M | FC | structural | LSCM | 24 hours | ↑ | Basal | Post |
| **Roppongi 2013** | DOI | 1 µM | NA | 5, 15 minutes | *in vitro* | Rats | Wistar | NR | HIP | structural PSD-95, SYN | LSCM | NR | — | Basal | - |
| **Rosa 2021** | Ketamine | 1 mg/kg | IP | Acute | *in vivo* | Mice | Swiss | F | HIP (ventral DG) | structural | Golgi stain | 45 minutes | —* | NSF | Post |
| **Sala 2022** | R-Ketamine | 10 mg/kg | IP | Acute | *in vivo* | Rats | Sprague-Dawley | M | PFC | structural | Golgi stain | 7 days | —* | Foot shock stress | Post |
| **Salort 2019** | Ketamine | 5, 15, 30, 150 mg/kg | IP | Acute | *in vivo* | Mice | Swiss albino IGS | M | Cortex | PSD-95 | western blot | 20, 50, 120 minutes | ↑(150mg/kg)@20, 50 min | Basal | - |
| **Sarkar & Kabbaj 2016** | Ketamine | 2.5, 5 mg/kg | IP | Acute | *in vivo* | Rats | Sprague-Dawley | M, F | mPFC | PSD-95, SYN; structural | western blot; LSCM | 3 days; 3 hours | ↑*@5 mg/kg for M, —*for F;  —*@2.5 mg/kg (except SYN, ↑*M) | Social isolation;  Basal | Post |
| **Seo 2020** | Ketamine | 100 μM | NA | 4-5 days | *in vitro* | Rats | Sprague-Dawley | NR | HIP | PSD-95; structural | western blot; immunostaining | NR | ↑*(100 μM) | DEX | Post |
| **Sinner 2011** | S-Ketamine | 3, 10, 25 µM | NA | 24 hours | *in vitro* | Rats | Wistar (embryonic) | NR | HIP | SYN | western blot | 24 hours | ↓(10, 25 µM) | Basal | - |
| **Strong 2017** | Ketamine | 2.5, 5 mg/kg | IP | 7 weeks (once weekly) | *in vivo* | Rats | Sprague-Dawley | M, F | NAc core vs. shell | structural | Golgi stain | 0.5 hours | @5 mg/kg: ↑NAc shell (M); ↑NAc shell, core (F); —2.5 mg/kg | Basal | - |
| **Strong 2019** | Ketamine | 0.5 mg/kg | IV | 4 weeks (3 days per week) | *in vivo* | Rats | Sprague-Dawley | M, F | NAc | structural | LSCM | 4 days | — | Basal | - |
| **Suarez-Santiago 2020** | Ketamine | 5, 10 mg/kg | IP | 5 days | *in vivo* | Mice | ICR | M | vmPFC, dStr, HIP (CA1) | structural | Golgi stain | NR | —vmPFC; ↓dStr; ↓CA1 | Basal | - |
| **Thelen 2016** | Ketamine | 3, 5, 10 mg/kg | IP | 21 days | *in vivo* | Mice | C57BL/6J | M, F | HIP | SYN | western blot | 24 hours | @10 mg/kg: ↑M; —F | Basal | - |
| **Tornese 2019** | Ketamine | 10 mg/kg | IP | Acute | *in vivo* | Rats | Sprague-Dawley | M | HIP (CA3) | structural | Golgi stain | 24 hours | ↑* | Unpredictable stress | Post |
| **Vutskits 2006** | Ketamine | 3.32-16.61 µM (acute)  0.03-3.32 µM (chronic) | NA | 4, 8 hours (acute); 24, 48 hours (chronic) | *in vitro* | Rats | Sprague-Dawley | NR | SVZ | structural | LSCM | 24, 48, 72, 96 hours | Dose-dependent ↓branching | Basal | - |
| **Vutskits 2007** | Ketamine | 3.32-66.45 µM (acute)  0.03-3.32 µM (chronic) | NA | 1, 8 hours (acute) 24, 48, 72 hours (chronic) | *in vitro* | Rats | Sprague-Dawley | NR | SVZ | structural | LSCM | 24, 48, 72 hours | Dose-dependent ↓branching | Basal | - |
| **Waller 2017** | Ketamine | 2 µM | NA | 1, 24 hours | *in vitro* | Rats | Sprague-Dawley pup | NR | HIP | structural; co-localization with SYN | LSCM | 0 hours | ↑@24 h for co-localization with SYN | Basal | - |
| **Wen 2019** | Ketamine | 10 mg/kg | IP | Acute | *in vivo* | Mice | C57BL/6 | M | HIP | PSD-95 | western blot | 3 days | ↑* | Unpredictable stress | Post |
| **Wesseling 2015** | S-Ketamine | 10 mg/kg | SC | Acute | *in vivo* | Rats | R150888 Lister hooded | NR | HIP, FC | SYN | SRM | 2 hours | ↓HIP; —FC | Basal | - |
| **Wu 2021** | Ketamine | 10 mg/kg | IP | Acute | *in vivo* | Mice | C57BL/6 | M, F | mPFC | structural | TPM | 2-72 hours | ↑spinogenesis@2-4 h; ↑spine density@12h-24 h | Basal | - |
| **Xu 2016** | DOI | 5 µg/0.5 µL | ICV | 4 weeks (once weekly) | *in vivo* | Rats | Sprague-Dawley | M | OFC | PSD-95; structural | western blot; Golgi stain | 11 days | ↓* | Chronic mild stress | Pre |
| **Xu 2020** | Ketamine | 15 mg/kg | IP | Acute | *in vivo* | Mice | C57BL/6 | M | dStr | structural | Golgi stain | 2 hours | ↑ | Basal | - |
| **Yang 2015** | Ketamine | 10 mg/kg | IP | Acute | *in vivo* | Mice | C57BL/6 | M | HIP (CA1, CA3, DG); mPFC (PrL, IL); NAc (core, shell) | structural | Golgi stain | 8 days | ↑*CA3, DG, —CA1; ↑*PrL,—IL; —*NAc | Social defeat stress | Post |
| **Yang 2016** | R-Ketamine | 10 mg/kg | IP | Acute | *in vivo* | Mice | C57BL/6 | M | HIP (CA1, CA3, DG), mPFC, NAc | PSD-95 | western blot | 8 days | ↑*CA3, DG, —CA1; ↑*PFC; —*NAc | Social defeat stress | Post |
| **Yin 2022** | Ketamine | 80 mg/kg/day for 5 days | IP | 5 days | *in vivo* | Mice | Kunming | NR | HIP | PSD-95 | western blot | 0, 5, 10, 24, 56 days | ↓@10, 24, 56 days | Basal | - |
| **Zhang 2015** | Ketamine | 10 mg/kg | IP | Acute | *in vivo* | Mice | C57BL/6 | M | HIP (CA1, CA3, DG), PFC, NAc | PSD-95 | western blot | 8 days | ↑*CA3, DG, —CA1; PFC; —NAc | Social defeat stress | Post |
| **Zhang 2018** | Ketamine | 10 mg/kg | IP | Acute | *in vivo* | Mice | C57BL/6 | M | HIP | PSD-95; structural | western blot; Golgi stain | 24 hours | ↑* | Unpredictable stress | Post |
| **Zhang 2019** | R-Ketamine | 10 mg/kg | IP | Acute | *in vivo* | Mice | C57BL/6 | M | HIP, mPFC | structural | Golgi stain | 3 hours | ↑* mPFC, CA3 and DG | Social defeat stress | Post |
| **Zhang 2020a** | Ketamine | bolus dose (40 mg/kg) and pump at 40–60 mg/kg/h for 3 hours | IV | Acute | *in vivo* | Rats | NR | NR | HIP (CA1, CA3) | structural | Golgi stain | 38 days (8 in uterus; 30 post-natal) | ↓ | Pregnant rat | 14 days of gestation (G14) |
| **Zhang 2020b** | Ketamine | 0.1, 1, 10, 100 µM;  10 mg/kg | NA  IP | 48 hours;  Acute | *in vitro; in vivo* | Rats | Sprague-Dawley | M, F; M | Cortex | structural | Golgi stain | 6 hours | ↓(100 µM); ↑(0.1, 1, 10 µM); ↑ (10 mg/kg) | Basal | - |
| **Zhao 2014** | Ketamine | bolus dose (40 mg/kg) and pump at 40–60 mg/kg/h for 2 hours | IV | Acute | *in vivo* | Rats | Sprague-Dawley | NR | HIP (CA3) | PSD-95; structural | western blot;  Golgi stain | 38 days (8 in uterus; 30 post-natal) | ↓ | Pregnant rat | 14 days of gestation (G14) |
| **Zhao 2016** | Ketamine | 144.2±4.6 mg/kg | IV | Acute | *in vivo* | Rats | Sprague-Dawley | NR | PFC | PSD-95, SYP; structural | western blot; Golgi stain | 8, 38 days (8 days in utero) | ↑PSD-95@38 days, ↓SYP; ↑spine density | Pregnant rat | 14 days of gestation (G14) |
| **Zhu 2022** | S-Ketamine | 20 mg/kg | IP | 7 days (once daily) | *in vivo* | Rats | Sprague-Dawley | M | HIP | PSD-95, SYN; structural | western blot; Golgi stain | NR | ↑* | Unpredictable stress | Post |

**Abbreviations:** ↑, increase; —, no change; ↓, decrease; ACC, anterior cingulate cortex; CA1 and CA3, cornu ammonis 1 and 3; CIC, chronic intermittent cold; CORT, corticosterone treated; DA, dopaminergic; DEX, dexamethasone treated; DG, dentate gyrus; dil, 1,1′-dioctadecyl-3,3,3,3′-tetramethylindocarbocyanine perchlorate; dmCP, dorsomedial caudate putamen; dlPFC, dorsolateral prefrontal cortex; dStr, dorsal striatum; DMT, N, N-dimethyltryptamine; DOI, 2,5-dimethoxy-4-iodoamphetamine; F, female; FC, frontal cortex; HIP, hippocampus; IL, infralimbic; IP, intraperitoneal injection; iPSC, inducible pluripotent stem cells; LPS, lipopolysaccharide; LSCM, laser-scanning confocal microscopy; LSD, lysergic acid diethylamide; M, male; mPFC, medial prefrontal cortex; NMRI, Naval Medical Research Institute; NA, not applicable; NAc, nucleus accumbens; NR, not reported; NSF, novelty-suppressed feeding; OFC, orbitofrontal cortex; PET, positron emission tomography; PFC, prefrontal cortex; PrL, prelimbic; PSD-95, postsynaptic density protein-95; SDCM, spinning disk confocal microscope; SRM, selected reaction monitoring mass spectrometry; SV2A, synaptic vesicle glycoprotein 2A; SVZ, subventricular zone; SYN, synapsin-1; SYP, synaptophysin-1; SYT, synaptotagmin-1; TEM, transmission electron microscopy; TPM, two-photon laser-scanning microscopy; TST, tail suspension test; vmPFC, ventromedial prefrontal cortex; WT, wild-type; *change observed in mice subjected to stress. Note: Spine density, branch number and arborization were considered as structural measures.

# Supplementary Table S2. Search Strategies for Scoping Review

| **Database** | **Results** |
| --- | --- |
| **PubMed**  ((((((((((((psilocybin[Title/Abstract]) OR (psilocin[Title/Abstract])) OR (lysergic acid diethylamide[Title/Abstract])) OR (LSD[Title/Abstract])) OR (N,N-dimethyltryptamine[Title/Abstract])) OR (DMT[Title/Abstract])) OR (mescaline[Title/Abstract])) OR (ibogaine[Title/Abstract])) OR (ayahuasca[Title/Abstract])) OR (2,5-Dimethoxy-4-iodoamphetamine[Title/Abstract])) OR (DOI[Title/Abstract])) OR (ketamine[Title/Abstract])) AND (((((((synaptic density[Title/Abstract]) OR (SV2a[Title/Abstract])) OR (synapsin[Title/Abstract])) OR (synaptotagmin[Title/Abstract])) OR (synaptophysin[Title/Abstract])) OR (PSD 95[Title/Abstract])) OR (dendrit*[Title/Abstract]))) | **478** |
| **Web of Science**  (TI=(((psilocybin OR psilocin OR "lysergic acid diethylamide" OR lsd OR n,n-dimethyltryptamine OR dmt OR mescaline OR ibogaine OR ayahuasca OR 2,5-dimethoxy-4-iodoamphetamine OR doi OR ketamine) AND ("synaptic density" OR sv2a OR synapsin OR synaptotagmin OR synaptophysin OR "psd 95" OR dendrit*)))) | **53** |
| **Scopus**  TITLE-ABS-KEY ( ( psilocybin OR psilocin OR "lysergic acid diethylamide" OR lsd OR n,n-dimethyltryptamine OR dmt OR mescaline OR ibogaine OR ayahuasca OR 2,5-dimethoxy-4-iodoamphetamine OR doi OR ketamine ) AND ( "synaptic density" OR sv2a OR synapsin OR synaptotagmin OR synaptophysin OR "psd 95" OR dendrit* ) ) | **132** |
| **EBSCO**  (psilocybin OR psilocin OR "lysergic acid diethylamide" OR lsd OR n,n-dimethyltryptamine OR dmt OR mescaline OR ibogaine OR ayahuasca OR 2,5-dimethoxy-4-iodoamphetamine OR doi OR ketamine) AND ("synaptic density" OR sv2a OR synapsin OR synaptotagmin OR synaptophysin OR "psd 95" OR dendrit*) | **52** |

# Supplementary Table S3. PRISMA-ScR Checklist

Preferred Reporting Items for Systematic reviews and Meta-Analyses extension for Scoping Reviews (PRISMA-ScR)

| **SECTION** | **ITEM** | **PRISMA-ScR CHECKLIST ITEM** | **REPORTED IN SECTION** |
| --- | --- | --- | --- |
| **TITLE** | | | |
| Title | 1 | Identify the report as a scoping review. | Title |
| **ABSTRACT** | | | |
| Structured summary | 2 | Provide a structured summary that includes (as applicable): background, objectives, eligibility criteria, sources of evidence, charting methods, results, and conclusions that relate to the review questions and objectives. | Abstract |
| **INTRODUCTION** | | | |
| Rationale | 3 | Describe the rationale for the review in the context of what is already known. Explain why the review questions/objectives lend themselves to a scoping review approach. | Introduction |
| Objectives | 4 | Provide an explicit statement of the questions and objectives being addressed with reference to their key elements (e.g., population or participants, concepts, and context) or other relevant key elements used to conceptualize the review questions and/or objectives. | Introduction |
| **METHODS** | | | |
| Protocol and registration | 5 | Indicate whether a review protocol exists; state if and where it can be accessed (e.g., a Web address); and if available, provide registration information, including the registration number. | Methods |
| Eligibility criteria | 6 | Specify characteristics of the sources of evidence used as eligibility criteria (e.g., years considered, language, and publication status), and provide a rationale. | Methods |
| Information sources* | 7 | Describe all information sources in the search (e.g., databases with dates of coverage and contact with authors to identify additional sources), as well as the date the most recent search was executed. | Methods |
| Search | 8 | Present the full electronic search strategy for at least 1 database, including any limits used, such that it could be repeated. | Methods |
| Selection of sources of evidence† | 9 | State the process for selecting sources of evidence (i.e., screening and eligibility) included in the scoping review. | Methods |
| Data charting process‡ | 10 | Describe the methods of charting data from the included sources of evidence (e.g., calibrated forms or forms that have been tested by the team before their use, and whether data charting was done independently or in duplicate) and any processes for obtaining and confirming data from investigators. | Methods |
| Data items | 11 | List and define all variables for which data were sought and any assumptions and simplifications made. | Methods |
| Critical appraisal of individual sources of evidence§ | 12 | If done, provide a rationale for conducting a critical appraisal of included sources of evidence; describe the methods used and how this information was used in any data synthesis (if appropriate). | Methods |
| Synthesis of results | 13 | Describe the methods of handling and summarizing the data that were charted. | Methods |
| **RESULTS** | | | |
| Selection of sources of evidence | 14 | Give numbers of sources of evidence screened, assessed for eligibility, and included in the review, with reasons for exclusions at each stage, ideally using a flow diagram. | Results |
| Characteristics of sources of evidence | 15 | For each source of evidence, present characteristics for which data were charted and provide the citations. | Results |
| Critical appraisal within sources of evidence | 16 | If done, present data on critical appraisal of included sources of evidence (see item 12). | - |
| Results of individual sources of evidence | 17 | For each included source of evidence, present the relevant data that were charted that relate to the review questions and objectives. | Results |
| Synthesis of results | 18 | Summarize and/or present the charting results as they relate to the review questions and objectives. | Results |
| **DISCUSSION** | | | |
| Summary of evidence | 19 | Summarize the main results (including an overview of concepts, themes, and types of evidence available), link to the review questions and objectives, and consider the relevance to key groups. | Discussion |
| Limitations | 20 | Discuss the limitations of the scoping review process. | Discussion |
| Conclusions | 21 | Provide a general interpretation of the results with respect to the review questions and objectives, as well as potential implications and/or next steps. | Conclusions |
| **FUNDING** | | | |
| Funding | 22 | Describe sources of funding for the included sources of evidence, as well as sources of funding for the scoping review. Describe the role of the funders of the scoping review. | Funding |

JBI = Joanna Briggs Institute; PRISMA-ScR = Preferred Reporting Items for Systematic reviews and Meta-Analyses extension for Scoping Reviews.

* Where *sources of evidence* (see second footnote) are compiled from, such as bibliographic databases, social media platforms, and Web sites.

† A more inclusive/heterogeneous term used to account for the different types of evidence or data sources (e.g., quantitative and/or qualitative research, expert opinion, and policy documents) that may be eligible in a scoping review as opposed to only studies. This is not to be confused with *information sources* (see first footnote).

‡ The frameworks by Arksey and O’Malley (6) and Levac and colleagues (7) and the JBI guidance (4, 5) refer to the process of data extraction in a scoping review as data charting*.*

§ The process of systematically examining research evidence to assess its validity, results, and relevance before using it to inform a decision. This term is used for items 12 and 19 instead of "risk of bias" (which is more applicable to systematic reviews of interventions) to include and acknowledge the various sources of evidence that may be used in a scoping review (e.g., quantitative and/or qualitative research, expert opinion, and policy document).

*From:* Tricco AC, Lillie E, Zarin W, O'Brien KK, Colquhoun H, Levac D, et al. PRISMA Extension for Scoping Reviews (PRISMAScR): Checklist and Explanation. Ann Intern Med. 2018;169:467–473. [doi: 10.7326/M18-0850](http://annals.org/aim/fullarticle/2700389/prisma-extension-scoping-reviews-prisma-scr-checklist-explanation).
